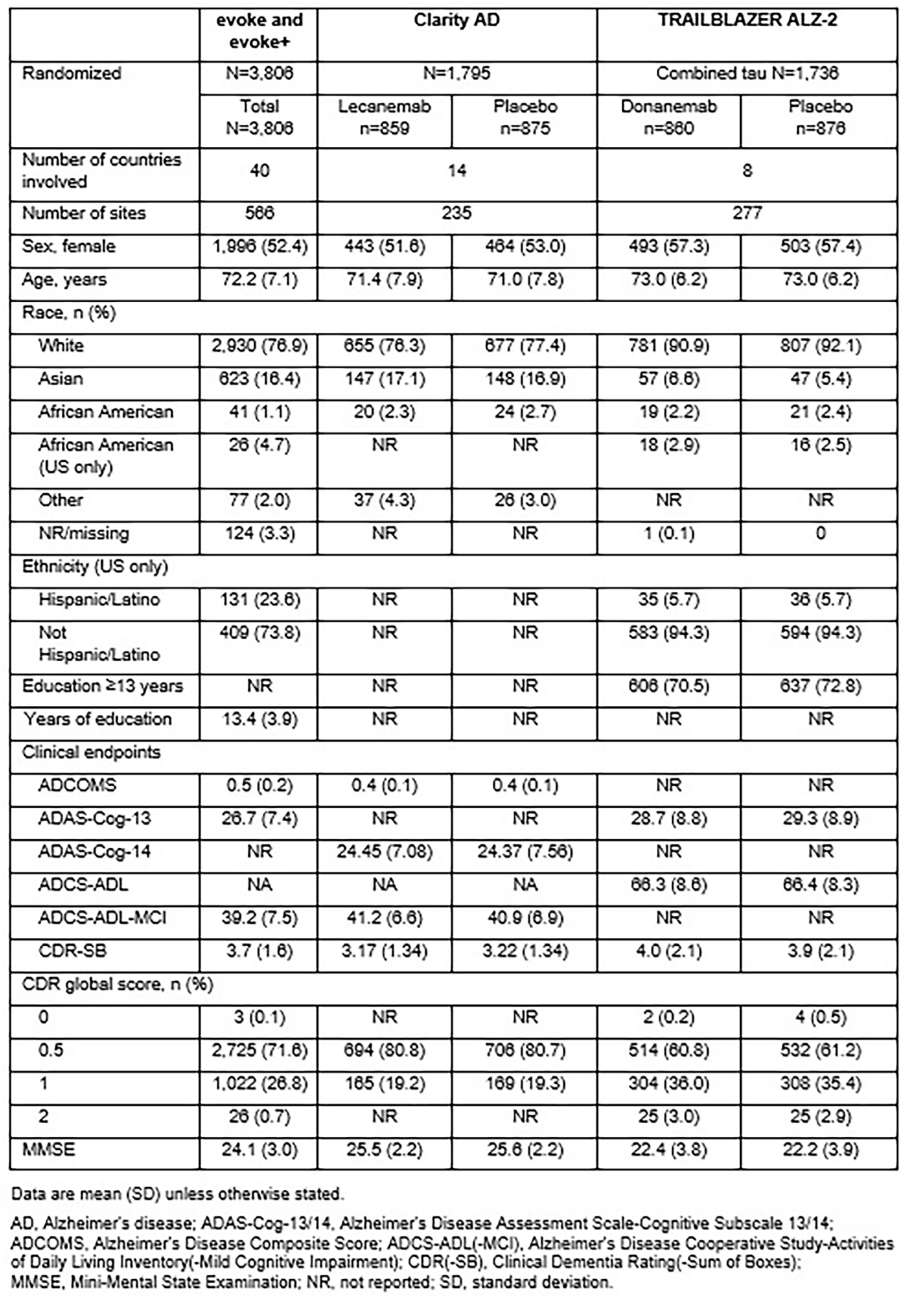# Comparison of eligibility criteria and baseline characteristics between the patient populations of evoke and evoke+, Clarity AD, and TRAILBLAZER‐ALZ‐2

**DOI:** 10.1002/alz.083684

**Published:** 2025-01-09

**Authors:** Howard H. Feldman, Philip Scheltens, Oskar Hansson, Mary Sano, Wiesje M. van der Flier, Lars Bardtrum, Peter Johannsen, Rose Jeppesen, Teresa Leon, Charlotte T. Hansen, Jeffrey L. Cummings

**Affiliations:** ^1^ University of California San Diego, La Jolla, CA USA; ^2^ EQT Life Sciences Partners, Amsterdam, 1071 DV Amsterdam Netherlands; ^3^ Lund University, Lund Sweden; ^4^ Icahn School of Medicine at Mount Sinai, New York, NY USA; ^5^ Amsterdam University Medical Center, Amsterdam Netherlands; ^6^ Novo Nordisk A/S, Søborg, 2860 Søborg Denmark; ^7^ Novo Nordisk A/S, Søborg Denmark; ^8^ University of Nevada, Las Vegas, NV USA

## Abstract

**Background:**

evoke and evoke+ are phase 3, randomized, placebo‐controlled trials currently investigating the glucagon‐like peptide‐1 receptor agonist semaglutide as disease‐modifying therapy (DMT) in persons with early Alzheimer’s disease (AD). How the evoke and evoke+ trial populations compare with other phase 3 programs for DMTs in early AD has not been described.

**Method:**

We compare the inclusion/exclusion criteria and baseline characteristics of the evoke/evoke+ trial populations with those of Clarity AD (lecanemab) and TRAILBLAZER‐ALZ‐2 (donanemab): two recent phase 3 trials assessing anti‐amyloid monoclonal antibodies in persons with early AD. A descriptive comparison is presented. Data cleaning of the randomized study populations of 3,806 participants in evoke/evoke+ is ongoing.

**Result:**

From Table 1, the four trials reported a total randomized sample of 7,337 participants. The broadest age range was in Clarity AD (50‐90 years vs 55‐85 years in evoke/evoke+ and 60‐85 years in TRAILBLAZER‐ALZ‐2). Race and ethnicity differed across trials, with the highest absolute number of non‐White participants in evoke/evoke+ (741 vs 402 in Clarity AD and 148 in TRAILBLAZER‐ALZ‐2; US Hispanic participation, 23.6% in evoke/evoke+ vs 11.4% in TRAILBLAZER‐ALZ‐2). Cognitive inclusion criteria also differed: evoke/evoke+ and Clarity AD required deficits in episodic memory at screening (based on WMS IV–Logical Memory II test and Repeatable Battery for Neuropsychological Status Delayed Memory Index, respectively). Mild cognitive impairment and mild AD dementia in evoke/evoke+ is defined by a Clinical Dementia Rating (CDR) global score of 0.5 and 1.0, respectively. All studies required participants to demonstrate amyloid positivity. TRAILBLAZER‐ALZ‐2 included tau pathology by positron emission tomography (PET) at screening, with low tau pathology the most frequent reason for study exclusion. The TRAILBLAZER‐ALZ‐2 population had more impairment on CDR‐Sum of Boxes, Mini‐Mental State Examination, and CDR global.

**Conclusion:**

All four studies targeted biologically defined early‐stage AD patients but differed in cognitive inclusion criteria. TRAILBLAZER‐ALZ‐2 differed from evoke, evoke+ and Clarity AD in having more impaired patients and using tau PET for staging. A higher number of non‐White participants were included in evoke, evoke+ and Clarity AD vs TRAILBLAZER‐ALZ‐2. Data for evoke and evoke+ are still subject to cleaning.